# Correct Use of Alkalies and Acids in Practice

**Published:** 1859-02

**Authors:** E. Andrews

**Affiliations:** Chicago, Illinois


### THE CHICAGO
### MEDICAL JOURNAL.

VOL. II. FEBRUARY, 1859. No. 2.

### ORIGINAL COMMUNICATIONS.

ARTICLE I.

### CORRECT USE OF ALKALIES AND ACIDS IN PRACTICE.

(Read to Cook Co. Medical Society.)

BY E. ANDREWS, M. D., CHICAGO, ILLINOIS.

The contest between vitalists and physicists is still maintained in some quarters to the injury of our science.

While the extreme vitalists maintain that common chemical affinities, such as those between alkalies and acids, have no existence in the living body, the ultra physicists claim that all the operations of life are mere modifications of the experiments in a chemist's laboratory. It is time to have done with this contest; but as the objections of one party or the other stand opposed to every possible doctrine on the relations of acids and alkalies to the tissues, the reader will indulge me in a few words to show that both sides are partly wrong. In the first place, living tissues are subject to all the mechanical laws, such as gravitation, momentum and impenetrability, also to the laws of levers, pulleys, valves, hydrostatics and pneumatics—for all these principles of inorganic nature are brought in and used as constant agents in physiologic life.

The subtler laws of the imponderables are equally efficient. Organic tissues have color, reflect light, and conduct heat and electricity as well as inorganic substances. All the laws of optics work with uufailing precision in the living eye, and the principles of acoustics do not fail when they are applied to the ear.

It is, however, in the department of chemistry that inorganic forces have been most strenuously denied access to living tissue. There is something incredible, however, in the assertion, that common chemical laws are entirely suspended during healthy life, if we consider that even the coarsest mechanical principles are not obliterated by vital force. If mechanic, hydrostatic, pneumatic, acoustic, optic and electric forces all work in the body, it is not difficult to believe that chemical affinity may have a place there also, and that an acid will saturate an alkali within the system as well as out of it. If facts are found pointing to this conclusion, vitalism should not declare it impossible.

On the other hand, the sweeping assertions of those who say that *all* living functions may be reduced to those few physical laws which are known to the natural philosopher and to the chemist, are presumption and folly. It is plain, beyond the necessity of argument, that living cells exercise functions which retorts do not imitate, and that laws are in operation in them which never have been, and probably never will be, reduced to the rules of the laboratory.

The position, therefore, will be assumed in this article, that there is no law which entirely prohibits the usual reactions of acids and alkalies in the system, and consequently the relations of these reagents to each other are properly the subject of examination and regulation by practical men in our profession.

The chief alkaline substances found in the human body are potash, soda, lime and ammonia. These are held in various states of combination with organic and inorganic substances, some of which are stable and neutral, as phosphate of lime in the bones; while others are feebly combined so as not to be neutralized, but to yield an alkaline reaction to the most delicate tests. Of the latter, the alkaline secretions of the salivary, lachrymal, hepatic and pancreatic glands are examples, to which may be added the blood, the semen, the secretions of serous and synovial membranes, the albumen around the ovum, and the serum of the areolar tissue.

Different alkalies are found in different parts, and their functions are various, but a somewhat general truth which meets us at the outset is this—*most of the alkalies promote a liquid form of protein compounds.* Thus, the liquid condition of albumen in the egg is dependent upon soda, and if the soda be neutralized by any acid, with certain exceptions hereafter to be mentioned, the albumen is immediately coagulated, or, in chemical language, is precipitated. The fibrin of the blood is held in solution, according to Dr. Richardson, by ammonia, which, being a volatile alkali, escapes when that fluid is exposed to the air, and precipitates the fibrin in the form of a clot, while the soda in the albumen of the serum not being volatile, retains that part of the blood in a liquid form. Hence the separation of the clot and the serum. An excess of ammonia will redissolve the clot, and even liquify the blood corpuscles themselves. This function of ammonia in the blood is proved by a variety of conclusive experiments on living animals, and is further shown by the fact, well known to every demonstrator of anatomy, that in the dead body the blood of the vena cava, which is surrounded by cavities secreting alkaline fluids, seldom coagulates until it is allowed to escape into the open air; while, in the very same subject, the blood of vessels which are imbedded among large muscles will form firm clots which may be drawn out of the veins like strings, and often will be found even in the arteries resisting the passage of injections. It seems to me that the explanation is simple: the large mass of blood in the vena cava does not favor the escape of ammonia, and the alkaline serum of the neighboring peritoneum certainly will have no tendency to neutralize it; but the juice of the muscles is acid, and immediately after death the action of endosmosis causes it to transude into all the nearest veins, effectually neutralizing the ammonia and consolidating the fibrin; hence the difference in the condition of the blood in the two cases.

From these facts, it seems to be a reasonable conclusion, that the alkalies (assisted by the acid phosphates) are the means used by nature to produce the liquid forms of protein.

If so, may it not be true that those pathological states which are marked by an excessive tendency to liquifaction, or, as the phrase is, "a breaking down" of the tissues, involve, as one essential condition, an excess of alkalies, especially of ammonia.

The following reasons lead us to this conclusion: First, this type of disease is often produced by putrid animal exhalations. Now, one of the earliest and most persistent products of animal decomposition is ammonia and its kindred compounds. It is evolved abundantly, not only from decaying flesh, but also from decomposing animal excretions of every kind, as sweat, urine, etc. Hence, it steams up in privies in combination with sulphuretted hydrogen; it arises in all filthy crowded places, as in ill-conditioned hospitals, camps, jails and ships; and it is formed in the human body itself, not merely in the last stages of diseases where the excretions are retained, but also in healthy subjects. Where patients are much exposed to this kind of influence, we find all the consequences which would naturally be expected. The muscular system failing in the amount of acid necessary to its activity, becomes feeble and prostrate, a violent effort at excretion of the alkalies often takes place in the form of erysipeloid infiltration, excessive suppuration, with pus so highly alkaline as actually to dissolve and abrade sound tissue over which it flows, or alkaline and profuse diarrhœa, or some other mode of elimination.

If the emunctories fail to get rid of the excess, the tissues at length begin to yield to the solvent power in ways perceptible to the eye. The blood corpuscles are liquidated; the walls of the small vessels often give way here and there, as in the hæmorrhagic type of confluent small pox, forming petechiæ; the solid tissues are obviously unable to consolidate protein enough to sustain their action; and the whole condition is said to be "low" and "typhoid."

Perhaps the true typical form of alkaline disease is erysipelas.

By this term I mean that malignant disease which is the terror of surgeons, and not the mere cutaneous eruption to which Prof. Meigs seems to restrict the name. The clearest and best definition of erysipelas is probably the one adopted and ably explained by Prof. Pitcher, of Detroit, who has given the subject the most masterly exposition extant.

Erysipelas is an inflammation, distinguished by a peculiar animal poison of great virulence, and capable of propagation by innoculation. It is essentially an aplastic disease, producing an abundant effusion of poisonous serum or pus, which is limited by no plastic lymph, but spreads indefinitely through the tissues. The analytic mind discovered at once that there are here two essential points, viz: the poison and the aplasticity, These two elements combined will constitute the disease; neither of them will do it alone.

It is to be observed here that erysipelas eliminates itself by the alkaline secretions, especially pus and serum; and it is singular to observe its indisposition to spread into tissues which oppose to it an acid reaction. The interstitial fluid of the muscles is acid: now, it is a matter of every-day observation that erysipelas will spread indefinately in the areolar tissue whose fluid is alkaline; it will cause this tissue to slough from whole extremities; it will burrow deep between the muscles, so that the dead tissue may be drawn out from deep recesses like shreds of wet tow,—but the muscles stand out round, granulating and uninjured. Thick muscles seldom or never slough from the direct effects of erysipelas. Is not this remarkable exemption due to the fact that the acid fluid of muscular tissue opposes an effectual barrier to the action of the alkaline poison?

The same difference, in a lesser degree, is observable in different persons. Erysipelas will not take its usual type in a strongly plastic system, or, what amounts to the same thing, in a system well supplied with acids. It is well known to practical anatomists that the poison of dissection wounds only exerts its effect fatally when it produces erysipelas, but that strongly plastic systems resist it, and present merely local inflammation. A striking instance illustrating this principle occurred in the experience of my respected preceptor, Prof. Pitcher, of Detroit, and which I take the liberty to repeat.

Prof. P., in company with a medical friend, made a *post-mortem* examination of the body of a patient who died of erysipelas. Both these gentlemen wounded themselves in the operation, and they were thus innoculated with the poison. The innoculation took effect in both, but in different ways. Prof. P.'s wound inflamed, but the progress was limited by plastic effusion, and a carbuncle was the result. The other gentleman, whose system seemed to have less plastic power, had erysipelas in the usual form, and died of its effects.

From these and a variety of other considerations we derive the conclusion that *malignant aplasticity requires, as one condition of its very existence, an excess of alkalies.* If active free acids (except the phosphoric) be present in abundance, this malignant pathological state cannot exist. The question also naturally suggests itself whether that mysterious condition called epidemic influence may not be, in many cases, merely a deficiency of acids and an excess of alkali in the system, produced by atmospheric or other general causes, which modify the chemistry of the living body. There is at such times the same aplastic type of disease, the same want of vital resistance to it, the same low grade of action, and the same tendency to solution of blood corpuscles and breaking down of tissues, which we observe in erysipelas. Indeed, so striking is this fact that Prof. Pitcher maintains that the malignant element in nearly all asthenic epidemics is identical with the peculiar poison of erysipelas; an opinion which contains much truth of the utmost practical value. It is probable that the asthenic epidemics nearly all derive their aplastic character from excess of alkalies; and we know that many of them are capable of propagating erysipelatous inflammation by innoculation, even when the original subject had no appearance to the eye of the latter disease.

The different proportions of acids and alkalies in the system give rise to several *types* or *grades* which run through almost every form of disease, and are easily recognizable in well-marked cases. The diagnostic marks of alkaline excess are *asthenicity* and *aplasticity*, while the signs of acid diathesis (except the phosphoric) are *sthenicity* and *plasticity*; while a due balance of these chemical vital agents will be manifested by the middle type of disease, such as simple inflammations, etc.

The differences thus indicated are traceable through every class of disorders. In peritonitis of the sthenic type and acid diathesis we have plastic effusion, glueing the intestines together; but in the alkaline diathesis we have purulent and other aplastic effusions. The plastic forms are pretty generally cured, but the aplastic variety is much more fatal, as in the malignant variety of puerperal fever. In pleurisy, if the diathesis be acid the result will be firm adhesions; but if excessively alkaline, purulent effusion will ensue. If bronchitis occur in an acid system it will be marked by the expectoration of pure mucus, by excessive development of the contractile fibres of the bronchi, occasioning spasmodic asthma, or by sudden metastases, such as are related by Bulkley in his rejected essay upon Rheumatic Bronchitis. But if the patient be under the influence of the alkaline diathesis, the inflammation will take the form of a catarrh, with puruloid expectoration, which often runs a rapid course.

The same kind of difference will be found to prevail in affections of almost every organ. When the acid diathesis is present, every disease takes the sthenic type; its effusions are plastic, suppuration is difficult, and all the phenomena approximate to that type of acid diseases—rheumatism. When the alkaline diathesis prevails, diseases are asthenic, effusions are aplastic, and tissues break down, and the disease approximates to the character of erysipelas.

If, for instance, the meninges of the brain be inflamed in an acid constitution, the membranes will be thickened, and the patient may fall into a chronic insanity, and in that state live out his days; but if the alkaline excess be present, the disease is of a different character. It runs a rapid course, the vessels relieve themselves speedily by an effusion of pus or serum, and the patient quickly dies.

If the case be scarlatina, in a plastic constitution, it will be of the sthenic grade and the simple variety; but if the alkaline diathesis prevail, the case will be asthenic, dangerous complications arise, and the patient is peculiarly liable to a malignant sore throat, which sloughs, ulcerates, and destroys life.

One of the most instructive of all the illustrations on this point is afforded by the two varieties of small pox. If this disease attacks a system well supplied with acids, and therefore strongly plastic, the disease is of the variety which we call "distinct." The pustules have firm boundaries of plastic effusion around them, which limits their spread, and separates them from each other; but if an excess of alkalies are present, the plastic effusion is not properly formed. The feeble boundaries are dissolved, the pus spreads irregularly in all directions, and contiguous pustules thus coalesce, constituting the confluent form of the disease. Every practitioner knows that the confluent character of the pustules is not merely owing to their close proximity to each other. I have seen distinct small pox where the pustules were as numerous and close together as in the confluent form, but the plastic power being strong, every pustule has a clear, smooth and circular border. If two adjoining pustules touched at their edges, they were separated by a sharp, fine red line for some time before coalescing. There was no loose spreading of one pustule to meet another.

Probably the best type of a purely plastic disease is found in rheumatism, which, in its connection with the acid diathesis, has been ably treated of by Fuller. In perhaps the majority of ordinary cases of disease, there is no remarkable excess of either alkaline or acid in the system, and in these cases of course no application of these doctrines can be made. In such a condition of the system we may have alkaline effusions in one part of the body and excess of the plastic deposits in another. The local influence in this case is sufficient to move the well poised balance. Thus, in the centre of a furuncle the mere weakening of the circulation resulting from the obstruction of the vessels causes the protein to assume the alkaline and liquid form of pus, while the circumference of the tumor continues in a firm plastic condition. Prof. Pitcher has published one well-marked case in which the same patient had rheumatism and erysipelas at the same time. I believe this combination to be very rare; yet, from the effect of local disturbances in the system, and where the general diathesis is not strongly acid or alkaline, I think both these diseases may co-exist even in portions of the body closely contiguous to each other.

The type of aplastic diseases may be found in erysipelas. The symptoms indicating this we have already discussed. That this aplasticity depends upon an excess of alkalies is shown by the chemical character of the remedies which have established the best reputation in that disease. The remedies which have been most approved in erysipelas are, almost without exception, such as have the power of neutralizing alkalies. The articles referred to are the following, viz: mur. tr. iron, tr. iodine, sulphate of iron, sulphate of copper, sulphate of quinine, nitrate of silver, bi-chloride of mercury, and mineral acid. Perhaps also we may add the favorite domestic remedy, the cranberry poultice.

Now let us look a moment at the reactions of these substances which have been approved by the experience of the profession as efficient in erysipelas. First, muriated tr. of iron. This substance contains the per-chloride of iron as its basis. It is extensively used as an internal remedy. The per-chloride of iron, whenever it meets with an alkali, either potash, soda or ammonia, immediately yields up its chlorine, and converts the base of the alkali to a chloride, destroying its solvent power in the tissues. It will be recollected that this is the substance sometimes used to form a clot in the cavity of an artery, and thus to plug it in lieu of ligating. It effects this object by neutralizing the ammonia which holds the fibrin of the blood in solution, and thus precipitating that substance. When given internally, it probably reduces the alkalinity of the blood so nearly to neutrality that that fluid can no longer exude alkaline; but on the contrary, the highly alkaline serum already effused into the inflamed areolar tissue begins, by common chemical laws, to recede again by endosmosis into the less alkaline blood.

Bi-chloride of mercury, as a local application, operates on the same principle. Its chlorine is feebly held and is readily given up to the alkaline bases wherever they meet.

Tr. of iodine, when it meets ammonia, neutralizes it by converting a part of it into iodate of ammonia, and the remainder into iodide of nitrogen, effectually destroying its alkaline qualities. When iodine meets potassa and soda, or their carbonates, it converts them into iodides and iodates of potassium and sodium. Hence the efficiency of this favorite remedy.

The sulphates of iron, copper and quinia all yield up sulphuric acid on contact with the alkalies.

Nitrate of silver, in like manner, yields nitric acid.

Mineral acids act directly of course upon the alkalies. The cranberry poultice seems to have some effect; and if so, it acts undoubtedly by virtue of the acid the berries contain.

The vegetable acids, when given by the mouth, are probably inert, because, as has been well proved by physiologists, they are digested and destroyed; and, as most of them hold a little alkali in solution, the alkali remains after the acids are destroyed, and increases the excess already existing.

If these principles embody any portion of truth, their application are of the utmost practical value. They furnish us the clearest possible rules for the use of the acids and alkalies in medicine.

In the confluent form of small pox, in erysipelas, and in the low aplastic types of all diseases, we should administer freely either mineral acids or such salts as will easily yield them up, or else such iodides and chlorides as part with their iodine and chlorine most readily. The following cases may illustrate the practical application of these ideas:

M. C. was attacked with small pox in apparently a virulent form. He never perfectly reacted. He sunk under the shock, and died before the eruption was fully out. His sister, a young lady, took the disease. Being of a more vigorous constitution, she resisted the shock better than her brother, but still the prostration was great, and the low typhoid condition gave me the gloomiest anticipations of the result. The pustules began tardily to make their appearance, but as thick as they could stand upon the skin. Anticipating a confluent case, I commenced the administration of mur. tr. of iron; dose, thirty drops every two hours; and continued until I believed the system was well saturated. The tone and vigor of her system improved greatly. The pustules were as thick as they could stand upon the skin, but they filled well, and though they literally crowded each other, yet each one preserved a clear, smooth outline. Only a very few, where they were the most crowded, coalesced with each other. The case convalesced favorably.

Mr. B. was daily occupied in the care of a friend who was on his death-bed with malignant scarlatina. There was much sloughing and inflammation of the sick friend's throat. Mr. B. had a small abraded pimple on the ala of the nose, which probably received poison by contact of the hand after lifting the sick man. Erysipelatous inflammation immediately commenced at the pimple and spread over half the face. I placed the patient at once on his back in bed, and kept on night and day ice-cold lotions of diluted muriatic acid. In twelve hours the disease began to subside, and at the end of thirty hours scarcely a trace of it was left. It may be of interest to state, that another attendant upon the same case of scarlatina was attacked with several abscesses on the sides of the neck and with a felon in the thumb.

In the light of these principles we see why erysipelas, in the form which is technically called absorption of pus, carries off so many surgical patients in badly kept hospitals. The decomposition of all nitrogenized animal matter gives rise at once to free ammonia. The perspiration, the urine, the fæces, the exhalations from the lungs, the pus, the blood, the serum,—in short, everything animal which remains a short time in the open air of a ward begins to putrify and exhale ammonia. This is inspired and absorbed by every patient present, and results in an alkaline diathesis that makes every surgical case one of danger.

Finally, the practice of giving ammonia as a stimulant in diseases which are marked by a tendency to liquifaction and breaking down of tissues, is irrational. The temporary stimulus which it communicates is desirable, but the absorption of it contributes to that excess of the alkalies which is already destroying life. It is to be feared that the indiscriminate exhibition of ammonia, in the last stages of malignant disease, very often hastens the coming dissolution, notwithstanding its temporary exciting power.

In conclusion, I will repeat the doctrines here maintained in the following formulæ:

1st. *Excessive plasticity and sthenicity indicates excess of acid or deficiency of alkali in the system; and considered as a diathesis, is to be combated by alkaline remedies.*

2d. *Aplasticity and asthenicity show a deficiency of acid or an excess of alkalies in the system; and this diathesis is to be combated with acids, or with such salts, chlorides or iodides, as neutralize the alkaline fluids.*

